# Smart Emergency Assistance System With Pre-Entry Access for Older Adults Living Alone: Simulation-Based Implementation Report

**DOI:** 10.2196/91542

**Published:** 2026-10-23

**Authors:** Juan Ying Chen, Kai Dong Lu, Yue Juan Cai, Yu Lian Li, Fei Yi Shen, Li Ji, Fang Yan Lu

**Affiliations:** 1 Department of Nursing, Tongxiang First People’s Hospital, Tongxiang, Zhejiang, China; 2 Department of Information Management, Tongxiang First People's Hospital, Tongxiang, Zhejiang, China; 3 Department of Hepatobiliary and Pancreatic Surgery, The First Affiliated Hospital, Zhejiang University School of Medicine, Hangzhou, Zhejiang, China; 4 Department of Nursing, The First Affiliated Hospital, Zhejiang University School of Medicine, 79 Qingchun Road, ShangCheng District, Hangzhou City, Zhejiang Province, China, Hangzhou, Zhejiang, 310003, China, 86 13505817318

**Keywords:** older adults living alone, home-based care, smart emergency system, preentry emergency access, intelligent access control, Internet of Things (IoT), MQTT, digital health, Message Queuing Telemetry Transport

## Abstract

**Background:**

Older adults living alone are at increased risk during medical emergencies, particularly due to delayed detection, information transmission, and physical access to the home. Digital health and Internet of Things (IoT)-based technologies offer opportunities to strengthen emergency response systems; however, evidence on the real-world implementation and performance of integrated home-based emergency assistance platforms remains limited.

**Objective:**

This study aims to describe the deployment and performance of a smart emergency assistance system for older adults living alone, and to evaluate key implementation outputs and performance indicators in a simulated environment.

**Methods:**

We implemented an emergency assistance system integrating the IoT, 5G technology, and mobile internet. An intelligent distress button served as the core user-facing device, while a hospital-based rescue management platform functioned as the central coordination hub. The implementation strategy focused on rapid signal transmission, automated multichannel notification, and precise location tracking to support home-based emergency response. System performance was assessed using predefined key performance indicators, including response time, notification success, positioning accuracy, and operational efficiency.

**Results:**

When the intelligent distress button was pressed continuously for at least 3 seconds, distress signals were transmitted to the hospital rescue management platform within 5 seconds. Notifications were automatically sent to the emergency medical services (EMS) and family contacts via SMS, with confirmation feedback completed within 10 seconds. The platform detected the GPS location of the person in distress with an error of less than 10 meters. Upon arrival of rescue personnel at the scene, system-enabled door unlocking was completed within 1 minute. The system demonstrated low hardware cost and low power consumption, with a distress trigger success rate of 97.78% (44/45) in simulated testing and 100% antimisoperation effectiveness.

**Conclusions:**

This implementation demonstrates the feasibility and scalability of a smart emergency assistance system for older adults living alone. By integrating emergency alerting, access control, and hospital-side coordination, the system supports rapid and efficient home-based emergency response and shows potential for scalable deployment in aging communities. However, several barriers to large-scale implementation remain, including incomplete integration with formal emergency dispatch systems, unvalidated hardware durability, and a lack of real-world user testing. Addressing these issues in future pilot deployments will be essential to transitioning from technical feasibility to operational readiness.

## Introduction

Population aging is a global demographic trend affecting countries worldwide. In recent decades, China has experienced particularly rapid demographic aging, with substantial shifts in population structure toward older age groups. According to data released by the National Bureau of Statistics, by the end of 2024, the population aged 60 years and older in China reached 310 million, accounting for 22% of the total population, while those aged 65 and above accounted for 15.6% [1]. Projections from the National Aging Office indicate that by the middle of this century, the older population in China will account for one-quarter of the global older population, marking a transition into a period of severe aging [2].

Among older adults, those living alone represent a particularly vulnerable subgroup. In emergency situations, older adults living alone often experience reduced physical coordination and impaired cognitive responsiveness, which may limit their ability to respond effectively to accidental injuries or acute medical events and can result in life-threatening consequences [3]. Emergency medical services (EMS), including ambulance dispatch via the national emergency number (120 in China), play a critical role in prehospital rescue and patient transport. However, a major practical barrier frequently encountered in real-world emergency response is the inability of rescue personnel to rapidly access the patient’s home upon arrival, often due to locked doors and the absence of family members or caregivers. To address this implementation gap, our team designed and deployed an emergency call and rapid response system tailored for older adults living at home who experience sudden critical illness. The system aims to facilitate timely distress signaling, coordinated emergency response, and rapid household access, thereby overcoming key technical and operational barriers that currently hinder effective prehospital rescue in domestic settings.

Specifically, the objectives of this implementation were to (1) establish a reliable emergency communication pathway between older adults, hospitals, and EMS; (2) enable timely and secure entry into the patient’s home during emergencies; and (3) evaluate system performance using predefined implementation-focused key performance indicators.

Previous studies have demonstrated the potential of digital health technologies, including wearable devices and Internet of Things (IoT) platforms, to support emergency detection and remote monitoring among older populations. However, many existing solutions remain limited to alert generation and lack integration with real-world rescue workflows, particularly regarding physical access to the patient’s environment. Evidence on the real-world implementation of integrated emergency assistance systems that bridge alerting, coordination, and pre-entry access remains limited.

This implementation report addresses this gap by focusing on system deployment, technical integration, and operational performance in a simulated clinical context, rather than solely on technological feasibility. Ma et al [4] conducted a survey covering 94 cities across 26 provinces in China and found that the vast majority of older adults and their family members support the use of emergency response systems; however, the single emergency response method used in home-based emergency services for older adults can easily lead to missing the golden rescue window. Currently, digital health technologies for emergency response in older adults have garnered increasing attention; nevertheless, most studies focus on technological development rather than systemic coordination and practical implementation solutions. Among the existing gaps, the implementation gap between information coordination and actual physical entry—specifically, the technical and operational mechanisms that enable emergency personnel to rapidly enter an older adult’s residence upon arrival—is a critical factor contributing to delays during the golden rescue window.

This study describes the design and simulated evaluation of an emergency call and rapid response system for acute and critical illnesses in older patients at home. A key technical limitation addressed by the system is the difficulty emergency responders face in gaining rapid entry into the patient’s home. By enabling emergency personnel to unlock the door promptly upon arrival at the patient’s doorstep, the system aims to avoid delays during the golden rescue window and thereby improve the success rate of resuscitation efforts. The principal practical contribution of this study is the provision of a prototype smart emergency assistance system that integrates distress alert triggering, multistakeholder synchronous response, remote physical access, and in-hospital data sharing. The system’s key performance indicators were validated in a simulated environment, offering an operational technical solution to the “physical access barrier” in emergency response for older adults living alone.

## Methods

### Implementation Context and Setting

This study adopts a 3D evaluation logic of “technical performance, process integrity, and safety and reliability.” Through system logs and observational measurements, we assessed the distress call success rate, transmission latency, positioning accuracy, unlocking time, full-process closed-loop completion, and fail-safe mechanisms to verify the system’s ability to operate stably and safely under simulated conditions. This implementation was conducted within a hospital-centered emergency response context, targeting older adults living alone in private residences. The system was designed to operate in coordination with hospital rescue management workflows and external EMS, supporting rapid response from distress initiation to on-site intervention.

The implementation aimed to address delays in emergency access and on-site entry that frequently occur when older adults experience acute medical events at home. By enabling rapid distress signaling, automated multiparty notification, and remote-controlled household entry, the system supported timely coordination from emergency initiation to on-site medical intervention.

### Intervention Description

The emergency assistance system comprised 4 core components: (1) an intelligent distress button located in the user’s home, (2) a smart key control box installed at the household entrance, (3) a cloud-based server equipped with Message Queuing Telemetry Transport (MQTT) services, and (4) a hospital rescue management platform serving as the central coordination hub (online home emergency call system). The overall system architecture is illustrated in Multimedia Appendix 1.

Once connected to the network, the intelligent distress button and the smart key box communicated with the server via the MQTT protocol. The hospital rescue management platform exchanged instructions and status information through the MQTT server, enabling real-time data transmission and processing for distress alerts and remote unlocking operations.

### Technical Architecture and Communication Framework

#### MQTT-Based Data Exchange

The MQTT protocol is a message transmission protocol based on TCP-IP networks, which adopts a publish-subscribe mode and features openness, simplicity, lightweight, and ease of implementation. It is particularly suitable for limited environments such as low bandwidth, unstable networks, embedded processors, and insufficient memory [5].

Message delivery reliability in MQTT is controlled by Quality of Service (QoS) levels. QoS Level 0 delivers messages at most once without guaranteed delivery; QoS Level 1 ensures messages are delivered at least once but may result in duplication; and QoS Level 2 guarantees exactly-once message delivery with the highest reliability [5]. In this implementation, QoS Level 2 was adopted to ensure that emergency distress messages were accurately delivered and processed by both the hospital rescue management platform and the smart key box terminals.

#### User Interface and Front-End Functions

The front-end of the hospital rescue management platform served as the primary interface for user interaction and emergency coordination. Core functions included user information management, IoT device binding, real-time device status monitoring, interactive message publishing and querying, and collaborative information processing.

Through the platform interface, family members were able to track the status of emergency alerts and device operation, while emergency personnel could receive distress notifications in real time, review basic patient information, and coordinate rescue actions across multiple departments.

#### Device Management Backend and External Integration

With the rapid proliferation of IoT applications, the number and diversity of connected devices continue to grow, posing significant challenges in device registration, status monitoring, data management, and coordinated operation. Heterogeneous IoT devices typically use different communication protocols and data formats, making centralized management and efficient information exchange crucial for ensuring system reliability and scalability—a requirement that necessitates an IoT management platform for unified control [6]. Based on this requirement, a centralized device management backend system was designed to integrate and manage all IoT devices, including smart emergency buttons and smart key boxes. The backend provided a unified management interface and API interface for device status monitoring, remote control, linkage control, and system maintenance.

The backend also supported data exchange with external institutions, such as emergency centers, enabling timely information sharing and coordinated response during emergency events.

#### Mobile-Based Interactive Tools

To enhance usability and accessibility, mobile-based interactive tools, including WeChat (Tencent) mini programs, were developed. These tools enabled users to receive emergency notifications and system reminders in real time. The mobile interface was seamlessly integrated with the smart IoT platforms, facilitating convenient access to system information and improving overall user experience.

#### System Deployment Environment

The system deployment environment included in-home IoT call buttons, IoT key boxes, internal and external network server zones, medical work areas, mobile devices used by users and family members, and 120 emergency center system server areas (planned for future integration; the current simulation used SMS-based notification instead of direct dispatch integration). The overall system composition is shown in Figure 1.

**Figure 1.**
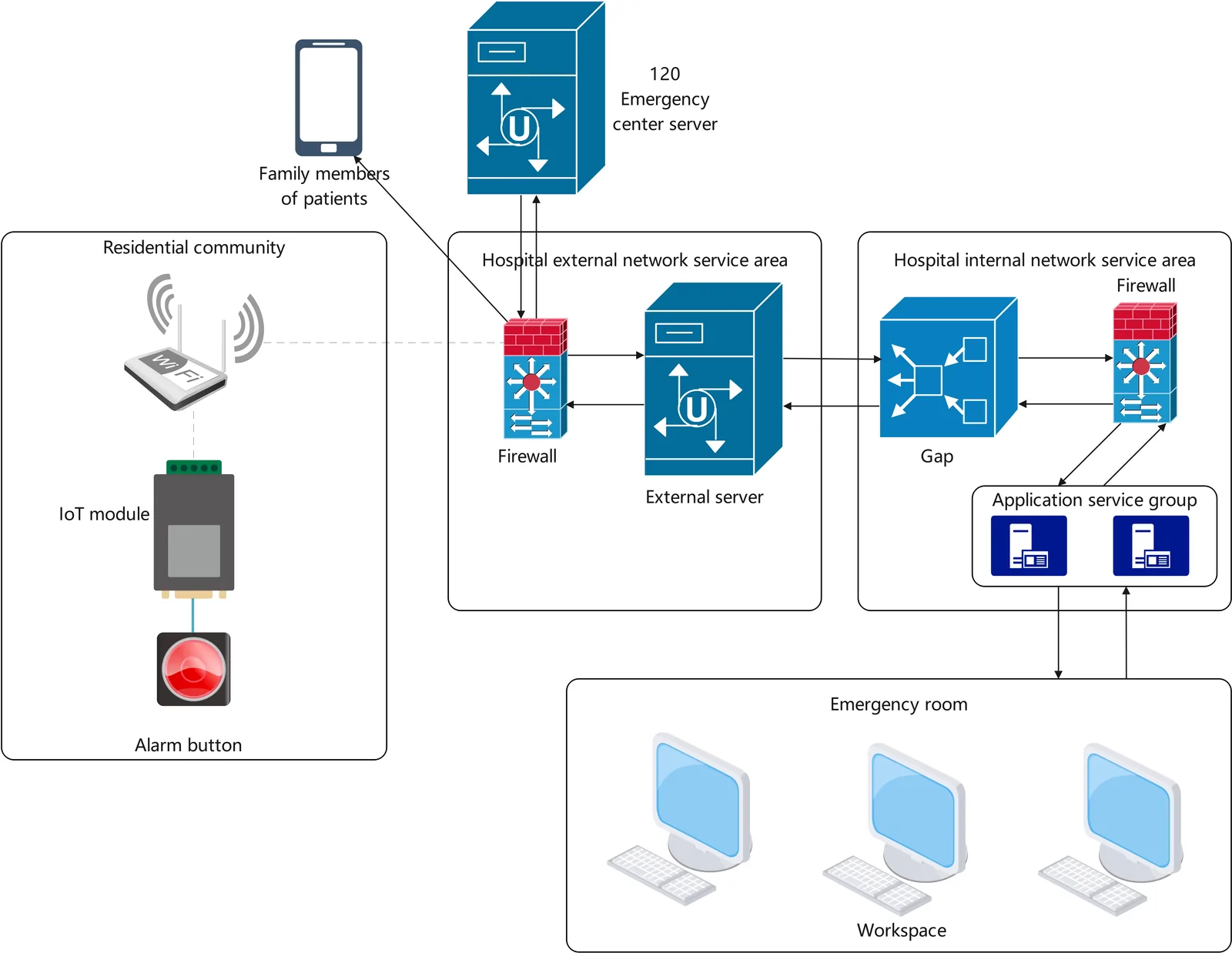
Deployment and system composition of the smart emergency assistance system. IoT: Internet of Things.

#### Security and Privacy Safeguards

Role-based access control is implemented following the principle of least privilege, with distinct roles defined for hospital dispatchers, 120 operators, family members, and other stakeholders. Remote unlocking of the smart key box requires trusted remote authorization. Mutual certificate-based authentication is enforced between devices and the platform. All operational records are stored in an immutable log and subject to regular auditing. Users sign informed consent agreements and are supported with the ability to withdraw consent. Transport Layer Security (TLS) encryption is applied to all data transmission. IoT modules in the system periodically report their status to the server to confirm communication availability. On the management side, abnormal devices are promptly flagged, and notifications are sent to administrators for follow-up action. For system availability, load balancing is introduced such that when a node fails, requests are automatically rerouted to available nodes. System stability is further supported by measures addressing network interruptions (local caching and retransmission), power failures (backup batteries), and false triggers (alert activation requires a long press of ≥3 seconds).

#### Hardware Components

IoT development board served as the core control units for both the intelligent distress button and the smart key box. ESP32 and ESP8266 microcontrollers were selected due to their low power consumption, high performance, high compatibility, and rich peripheral interfaces, making them very suitable for IoT applications [7,8]. These controllers processed user input signals and communicated with smart IoT platforms. The circuit design of ESP32 is shown in Figure 2.

**Figure 2.**
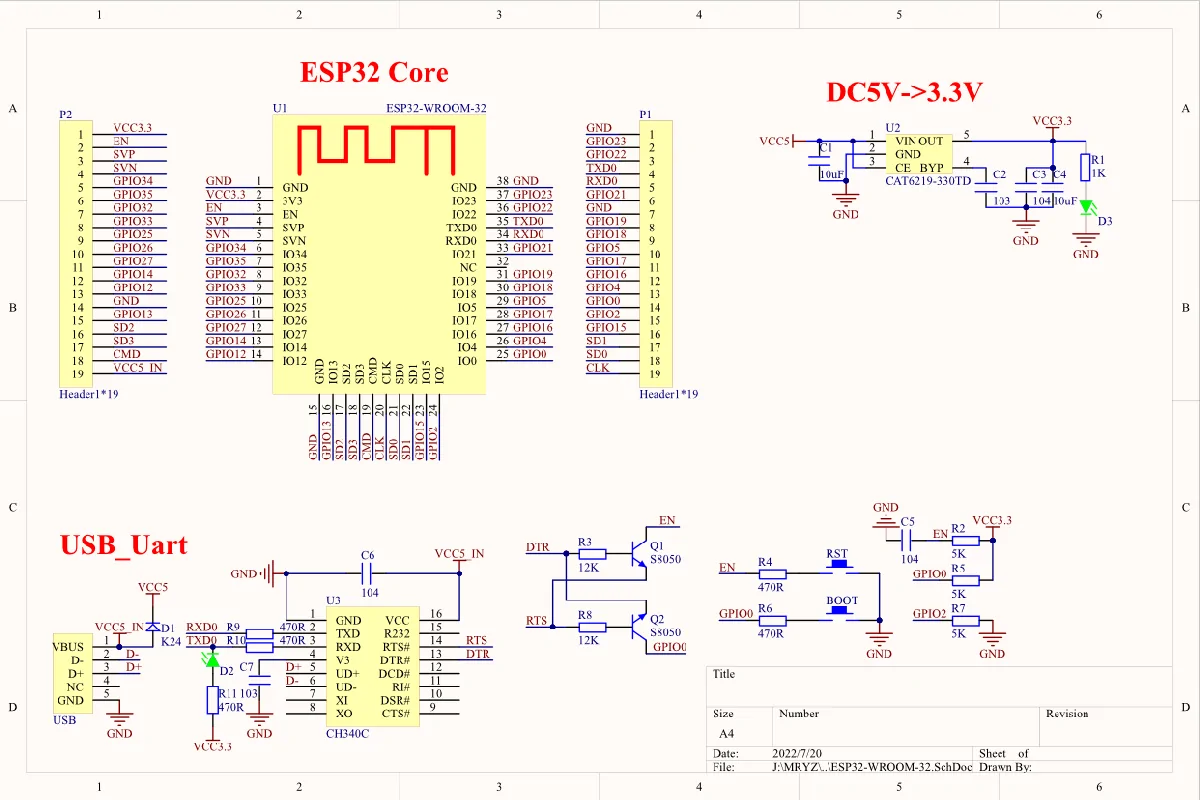
Hardware implementation of the ESP32-based emergency call device.

#### Intelligent Distress Button

The intelligent distress button functioned as the primary user-facing interface and was designed to be intuitive and easy to operate. A regular hexagonal button with a side length of 35 mm was selected to accommodate older users [9]. To address concerns regarding older adults’ digital literacy and prevent false alarms, the button adopted an antimisoperation design: a single click is ineffective, and users must press and hold the button for at least 3 seconds to trigger an alert. Stable electrical connections and circuit protection measures, including current limiting and electrostatic discharge protection, were incorporated to ensure reliability [10]. Visual indicators, such as LED lights, were used to display device status.

#### Smart Key Box and Electromagnetic Lock

The smart key box was constructed from durable materials to withstand environmental exposure and potential external damage. It supported multiple installation methods, including wall mounting and peephole installation.

An electromagnetic lock served as the access control mechanism, allowing authorized remote unlocking during emergency situations. The system used a 5V 4-wire electromagnetic lock controlled via a relay driven by the ESP8266 microcontroller [11]. A high-level control signal of approximately 0.5 seconds triggered the unlocking mechanism. Lock status feedback signals were monitored in real time to confirm successful unlocking and ensure operational safety.

#### Power Supply and Energy Management

Flexible power supply solutions were adopted based on device function and usage scenarios. Smart key boxes requiring long-term stable operation were powered by portable power modules or large-capacity lithium batteries (recommended capacity of ≥5000 mAh). Intelligent distress buttons with low power consumption requirements used coin cell button batteries (eg, CR2032) or rechargeable lithium batteries. Voltage monitoring functions were incorporated to alert users when battery levels were low, supporting continuous and reliable system operation.

#### Evaluation Design and Testing Environment

To evaluate the feasibility and operational performance of the emergency assistance system, functional and integration testing was conducted under simulated real-world conditions. The evaluation covered end-to-end emergency workflows, including distress activation, data transmission, platform response, notification delivery, positioning, and remote unlocking.

The experimental platform and configuration are as follows: the distress button is made using ESP32 series modules as the core, the MQTT message broker uses EMQX platform (Hangzhou EMQ Technologies Co., Ltd.), the server uses an in-house virtual machine running Microsoft Windows Server 2012, with a specific configuration of an 8-core processor, 4G memory, a system database of MySQL 5.7 (Oracle Corporation), a keybox built using ESP8266 series modules and electromagnetic locks, the platform system development tool is Eclipse (Eclipse Foundation), the IoT board development tool is Thonny (Aivar Annamaa), the firmware is MicroPython, and the development computer OS is Windows 11.

#### Participant Selection

Hospital medical staff were recruited as volunteers through an internal hospital announcement. Inclusion criteria were (1) currently employed medical staff familiar with emergency procedures; (2) voluntary participation with signed informed consent; and (3) no severe visual or hearing impairment that would prevent normal operation of the smart emergency button. A total of 5 volunteers (3 females, 2 males; age range 28‐45 years) were enrolled, all of whom played the role of the person initiating the distress call. Other response roles—including hospital platform operators, 120 simulated dispatchers, and simulated family contacts—were performed by research team members and hospital medical staff.

#### Testing Duration and Scenarios

System simulation testing was completed within a single day, with a total duration of approximately 4 hours. Three types of scenarios were designed: (1) active long press of the emergency button (≥3 seconds), (2) antimisoperation testing (short press <3 seconds), and (3) consecutive distress activation (10 minutes of continuous testing, with 3 triggers within a 10-minute window). Each volunteer completed all 3 scenarios sequentially, performing 3 tests per scenario, resulting in a total of 9 simulated distress events per participant. For each distress event, the entire process from button activation to key box unlocking was quantitatively recorded using system logs and structured observation forms. The average duration per event was approximately 2 minutes.

#### Evaluation Criteria

Prespecified key performance indicators were used to assess system effectiveness: (1) distress trigger success rate: the proportion of long presses (≥3 seconds) resulting in successful alert reception by the platform, required to be ≥99%; (2) transmission latency: the time interval from button activation to “received” timestamp in the hospital platform log, required to be ≤10 seconds; (3) lock release completion time: the duration from rescue personnel’s unlock request to key box release, required to be ≤1 minute; and (4) antimisoperation effectiveness rate: the proportion of short presses (<3 seconds) that did not generate an alert, required to be 100%. All indicators were dual-recorded using system backend log timestamps and on-site observation forms.

#### Observation and Recording

Two research team members (Lei Feng Jiang, Hui Qing Chen) were present throughout the testing process. They used a structured observation form to document each button operation type, platform feedback status, lock release execution, and any anomalies. Observation form data were cross-checked against system logs by 2 independent reviewers to ensure consistency. Regarding user privacy and security, all system data were stored on servers on the hospital premises, and data transmission was encrypted through interfaces. To ensure comprehensive data collection and support transparent evaluation, 3 types of data sources were used in this study: (1) system backend logs: these recorded key performance indicators including smart emergency button trigger time, response confirmation time, unlock operation logs, and data transmission integrity; (2) simulated emergency scenario observation form: this structured form was used to document the complete distress call process conducted by the 5 medical staff volunteers in the 15-square-meter simulation site; and (3) device stability test records: these provided statistics on system interruptions, functional failures, and false alarm/missed alarm counts during continuous operation.

#### Simulated Clinical Implementation Procedures

The system was evaluated in a simulated clinical implementation setting to assess its feasibility, stability, and workflow integration under conditions approximating real-world home emergency scenarios. Following ethical review and approval, a room with an area of 15 square meters was selected as the simulation site, and the system was installed according to the design requirements. This study was conducted as a pilot project. After passing simulation testing, it was put into clinical validation.

As described above, 5 medical staff members were recruited as volunteers to participate in simulation emergency scenarios. During the implementation period, the research team observed the full emergency response workflow, including distress signal triggering, system connectivity monitoring, alert transmission and confirmation, key box unlocking, and information access via the platform. All simulated emergency events progressed through the predefined workflow without system interruption or functional failure.

### Ethical Considerations

This study was reviewed and approved by the Tongxiang First People’s Hospital Ethics Committee (approval number 2025-082-01).

## Results

### Implementation Outputs and System Performance

#### End-to-End Emergency Response Performance

System-level testing demonstrated that the emergency assistance system was able to support home-based emergency response during simulated acute medical events. Regarding the average time from emergency trigger to successful pre-entry activation, when the smart emergency button is pressed and held for 3 seconds or longer, the system transmits the distress signal to the hospital rescue management platform within 5 seconds, while simultaneously automatically notifying the 120 emergency dispatch center and the patient’s family members, ensuring that the distress message is received and acknowledged within 10 seconds. Upon receiving the emergency alert, the platform accurately detects the GPS location of the person in distress with a positioning error of less than 10 meters. After rescue personnel arrive at the scene, the system completes the remote unlocking process within 1 minute. From the moment of distress activation to receipt and confirmation of the emergency signal by the platform, the complete communication and feedback process was completed within 18 seconds. The system supported a one-click quick distress operation, enabling emergency notification and coordination immediately after activation. Following confirmation by medical personnel, the system enabled remote unlocking of the household entry point within 1 minute of rescue personnel arrival, thereby reducing delays during the critical emergency response window.

#### Emergency Button Activation

When the emergency button was pressed continuously for at least 3 seconds, the ESP32 controller detected the input and provided visual feedback via a flashing indicator light. In a subset of stability tests (10 consecutive activations), the device demonstrated a 100% triggering success rate, while the overall rate across all 45 scenario tests was 97.78%. An example of the emergency button activation process is shown in Multimedia Appendix 2.

#### Distress Signal Transmission

Once activated, the emergency button automatically transmitted a distress signal to the backend server via MQTT. The server receives and parses the message and generates automated notifications to on-duty medical staff via text messages. Distress records were simultaneously logged and displayed in the rescue management platform for further processing. An example of a distress notification message is shown in Multimedia Appendix 3.

#### Distress Information Management

The rescue management platform provided a centralized interface for managing incoming distress alerts. Pending alerts were displayed with detailed information, including distress type, distress time, button name, detailed address, alarm reception time, alarm confirmation time, alarm confirmation person, alarm processing status, and so on (Figure 3).

**Figure 3.**
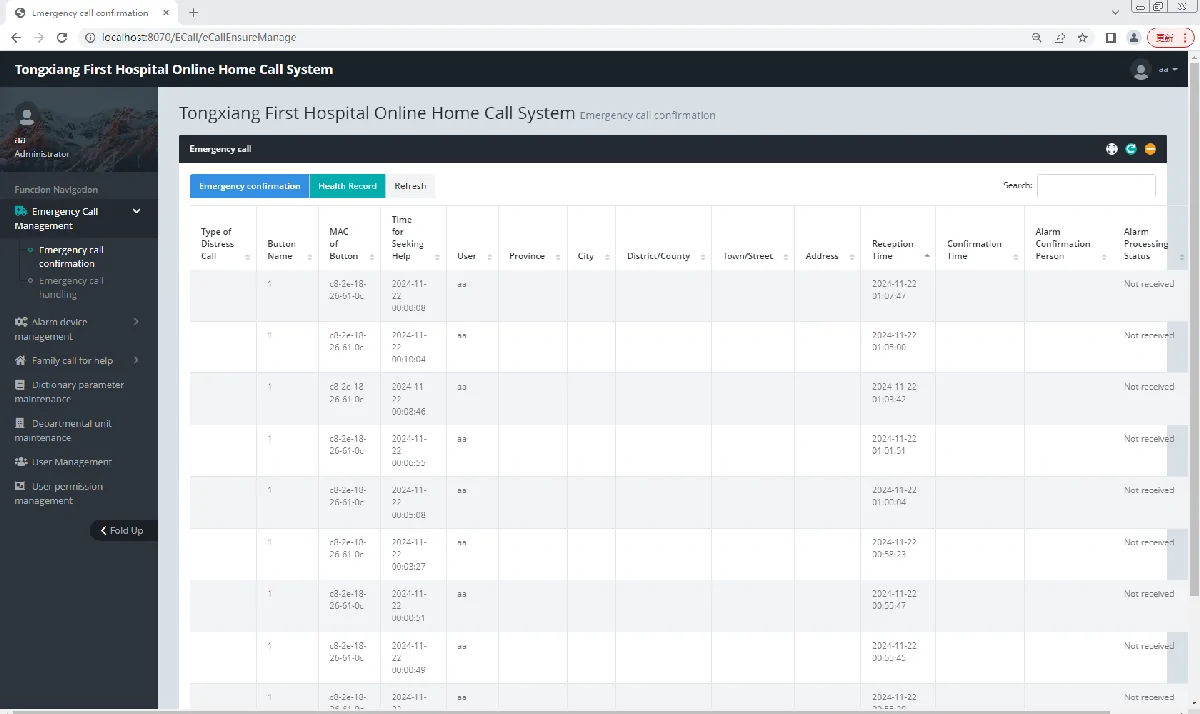
Management platform interface displaying system-generated emergency alert information.

Select the corresponding distress message line, click the “distress confirmation” button above to confirm the distress message or cancel the distress call according to the actual situation. The confirmation distress message interface is shown in Figure 4. Medical personnel were able to confirm or cancel distress alerts based on real-time assessment. Upon confirmation, the system automatically initiated notification to the 120 EMS dispatch and synchronized alert information with the EMS system (Multimedia Appendix 4). The platform also allowed authorized staff to access integrated hospital health records, including historical diagnosis and treatment information, medication records, surgical records, and examination records, to support informed decision-making (Multimedia Appendix 5).

**Figure 4.**
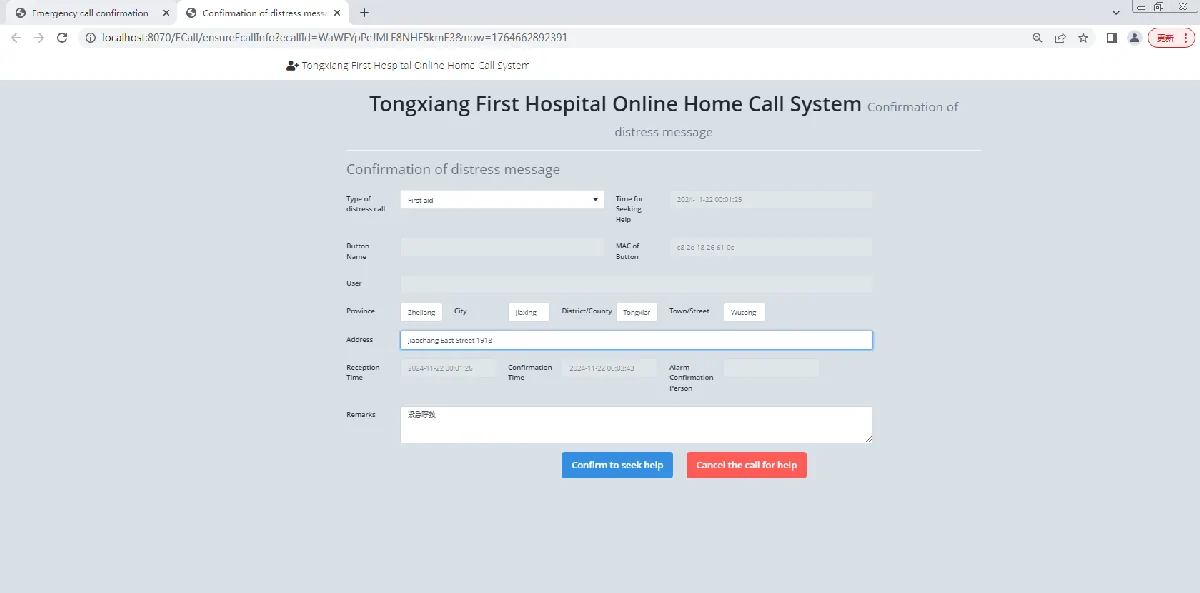
Emergency alert confirmation interface in the management platform.

### Device Management and Monitoring

A device management module enabled administrators to initialize, bind, and maintain intelligent distress buttons and smart key boxes. Device-level information, including device identifiers, firmware versions, network status, and installation records, was centrally managed. The platform displayed real-time online status of registered devices using color-coded indicators, facilitating proactive maintenance and fault detection (Figures 5-7).

**Figure 5.**
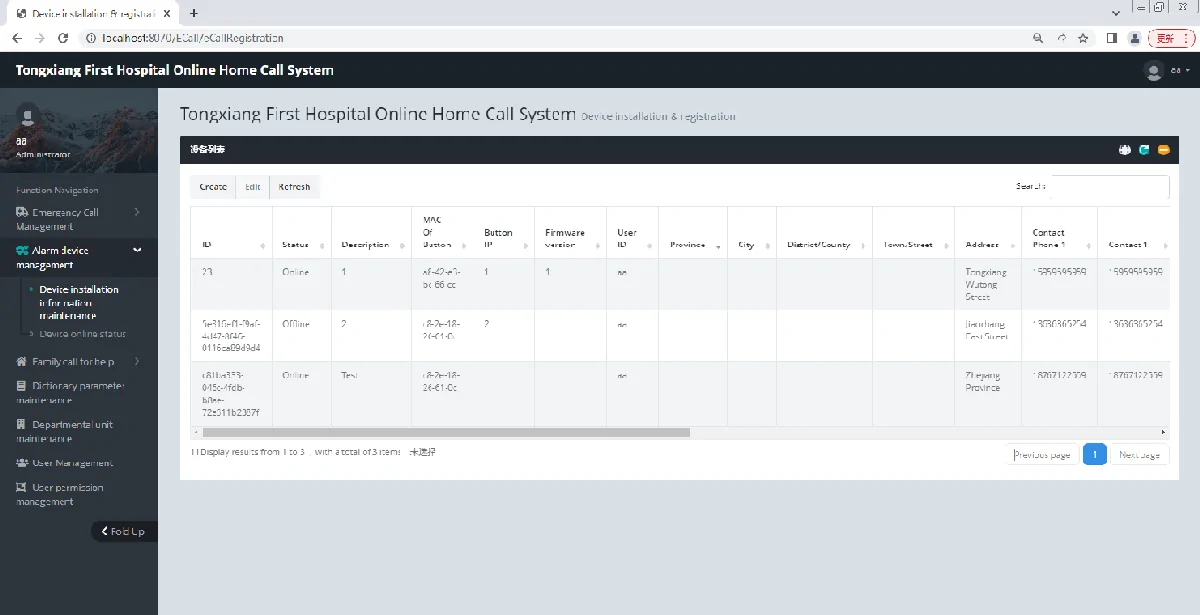
Interface displaying device installation information.

**Figure 6.**
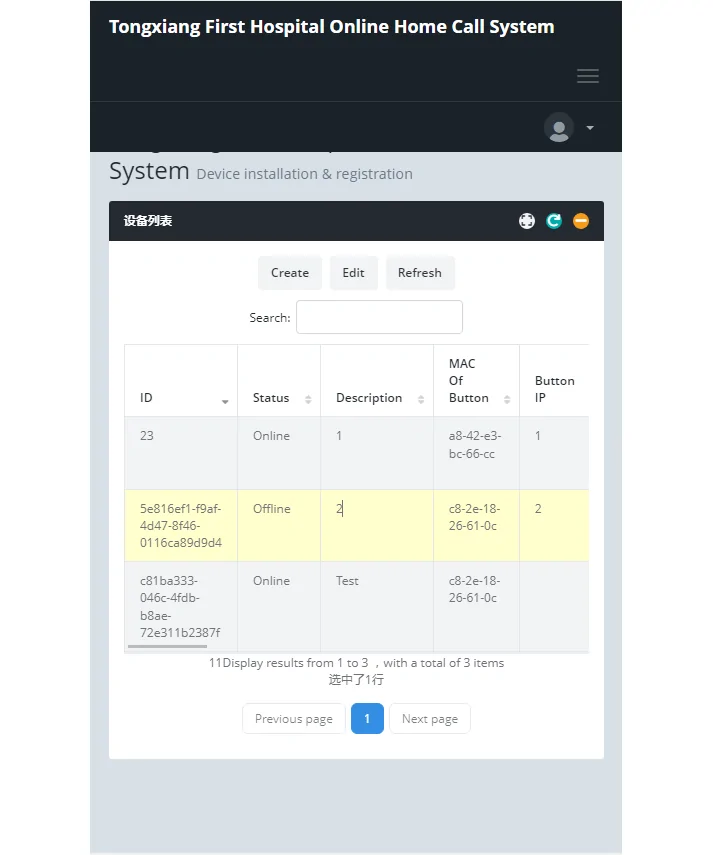
Interface for managing emergency button installation and configuration.

**Figure 7.**
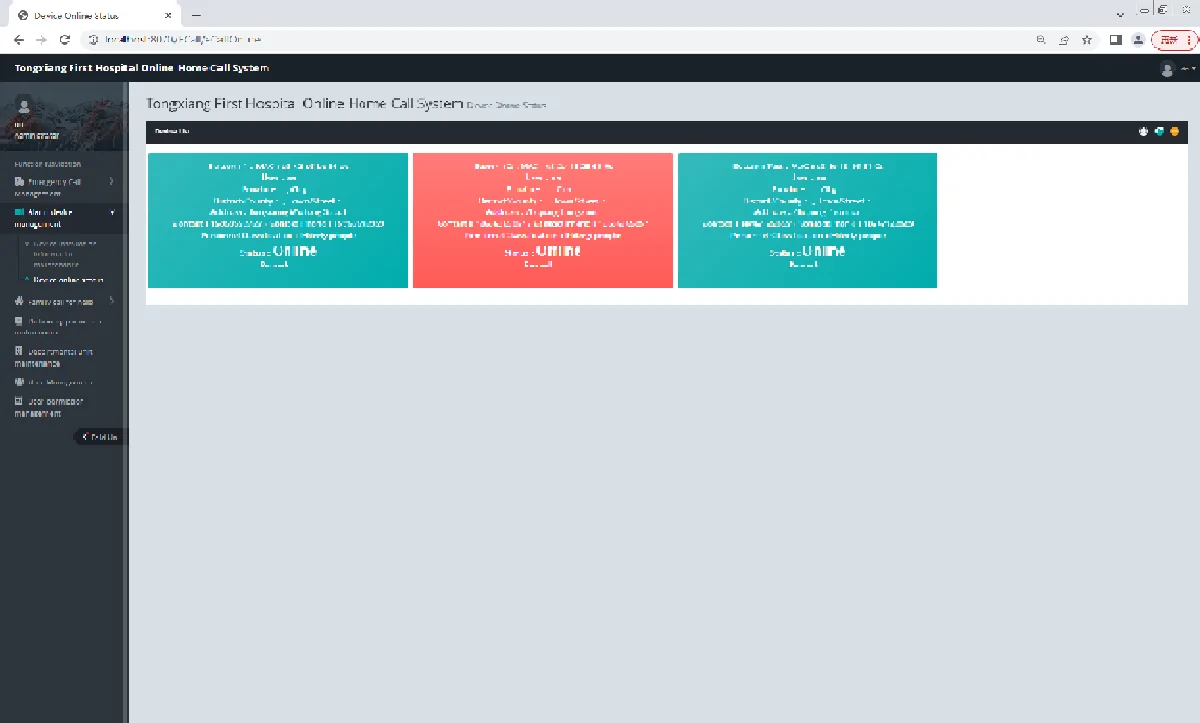
Interface showing real-time device connectivity status.

### Remote Lock Control and Household Access

#### Overview

When the smart key box (electromagnetic lock control board) receives a home distress request confirmed and forwarded after distress confirmation by medical staff, the smart key box receives a remote unlock command. The key box emitted a visible SOS Morse-code flashing signal and automatically released the electromagnetic lock, enabling rapid access for rescue personnel or nearby responders. A functional example of the lock control module is shown in Figure 8.

**Figure 8.**
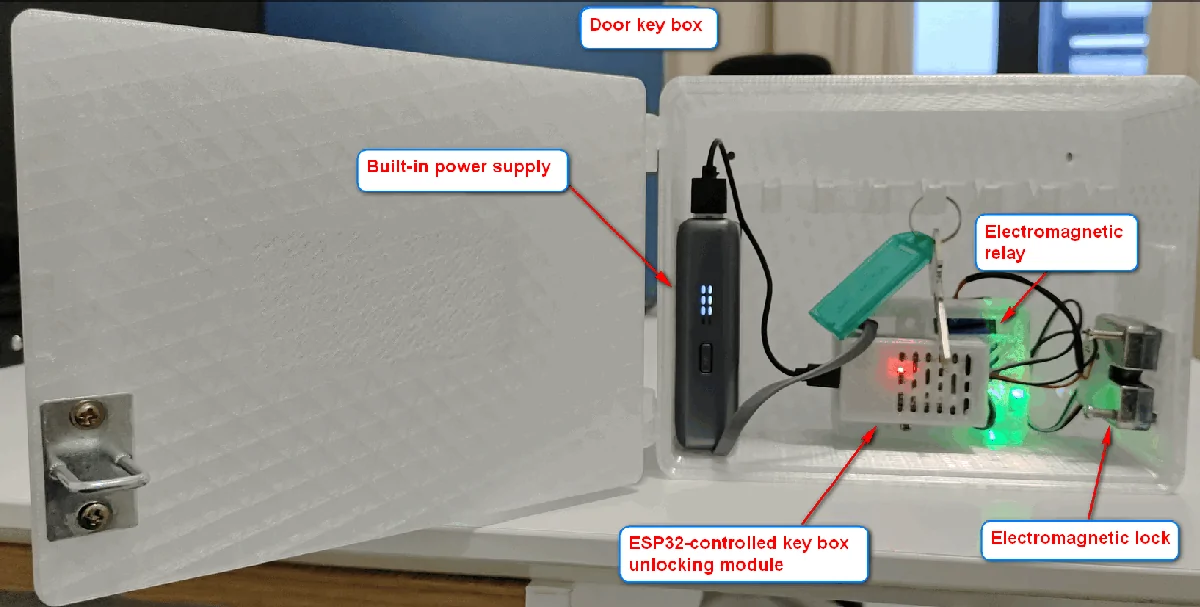
Example of the lock control module in operation.

#### Observed Outcomes

The system demonstrated stable and timely operation across all key stages of the emergency response workflow. A total of 45 application scenario tests were completed, consisting of 5 volunteers each performing 3 types of scenarios—active long press (≥3 seconds), antimisoperation test (short press＜3 seconds), and consecutive distress activation (3 triggers within 10 minutes)—with 3 repetitions per scenario. All tests were conducted in a 15-square-meter room equipped with the smart emergency assistance system. In a subset of device stability tests (10 consecutive activations), the triggering success rate was 100%. Across all 45 simulated scenario tests, the overall success rate was 97.78% (44/45), with the single failure attributed to a 3D-printed housing precision issue, which prevented successful activation in a specific instance; the issue was subsequently resolved by optimizing the button printing model. No system interruptions were observed during testing. The system achieved a response time within 10 seconds, 100% antimisoperation effectiveness, GPS positioning accuracy within 10 meters, and a 100% successful unlocking rate within 1 minute.

Upon receiving the emergency alert, the platform successfully detected the GPS location of the person in distress with a positioning error of less than 10 meters. Subsequently, the platform issued a remote unlock command, triggering the electromagnetic lock automatically. Household entry was completed within 1 minute in all test scenarios.

In addition, following patient admission, integration with the hospital information system allowed authorized medical staff to access historical health records, including prior diagnoses and treatment information. This functionality supported timely clinical decision-making and facilitated preparation for subsequent in-hospital emergency care.

#### User Interface Evaluation

The older adult–facing interface requires only a single long press (≥3 seconds). Separately, the health care–facing platform interface was evaluated by 5 medical staff volunteers—4 of them rated the interface design as user-friendly and the functions as convenient, and one suggested that more features could be added to support medical decision-making.

Formal usability testing with older adult users has not yet been conducted.

#### Feasibility in a Simulated Implementation Setting

The simulated implementation demonstrated that the system could be effectively integrated into an institutional emergency response workflow. All simulated emergency events progressed through predefined procedures without system interruption or functional failure.

Overall, the simulated clinical implementation demonstrated that the system could be integrated into an institutional emergency response workflow and support coordinated, time-sensitive actions across multiple roles. No major implementation barriers were identified during the simulation period, supporting the feasibility of the system for home-based emergency assistance in the older population living alone. The overall business process of system design [12] is shown in Figure 9, and the user cases are shown in Figures 10 and 11. A detailed step-by-step implementation workflow is provided in Multimedia Appendix 6.

**Figure 9.**
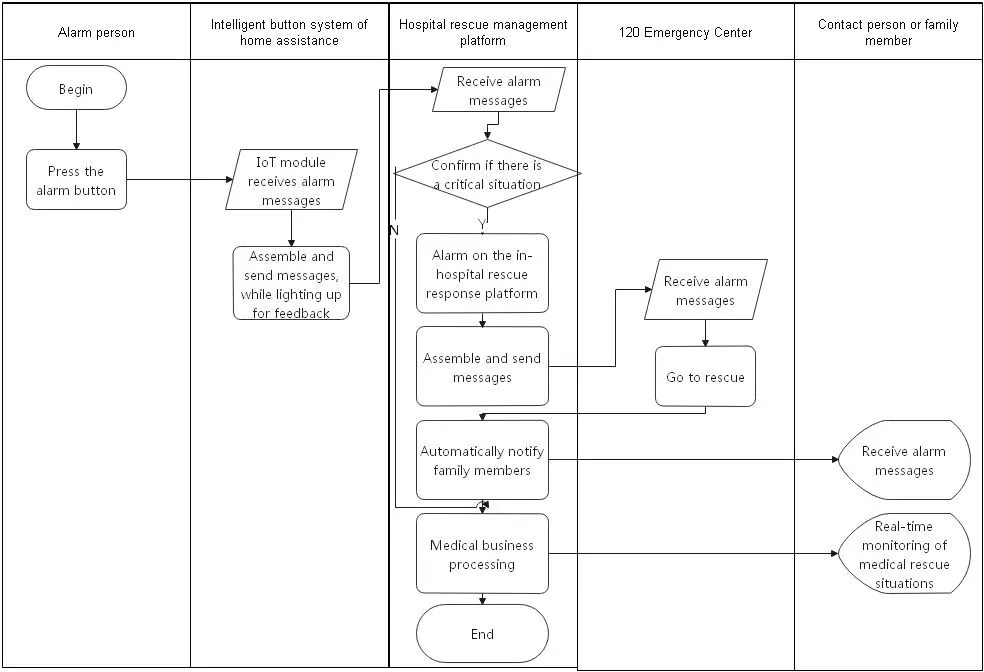
Overall system workflow of the emergency assistance implementation. IoT: Internet of Things.

**Figure 10.**
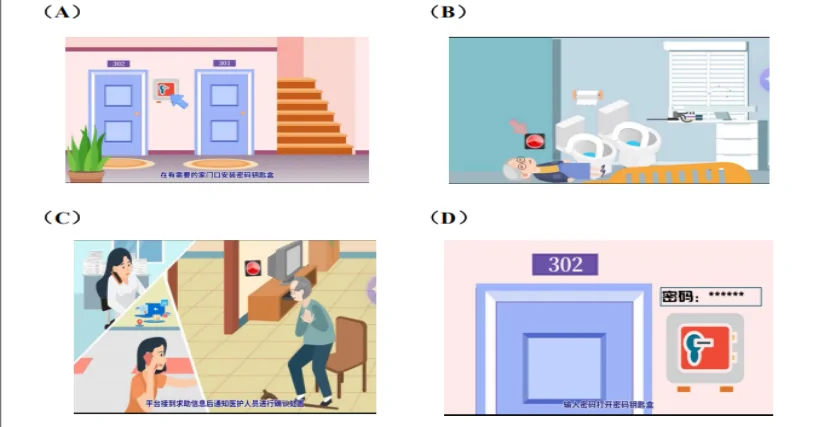
App scenario of the emergency assistance and pre-entry access system. (A) Installation of the smart key box at the household entrance. (B) Emergency call activation by sustained button press (≥3 seconds). (C) Distress alert reception and processing by the rescue management platform. (D) Authorized household access by rescue personnel using a dynamic access code.

**Figure 11.**
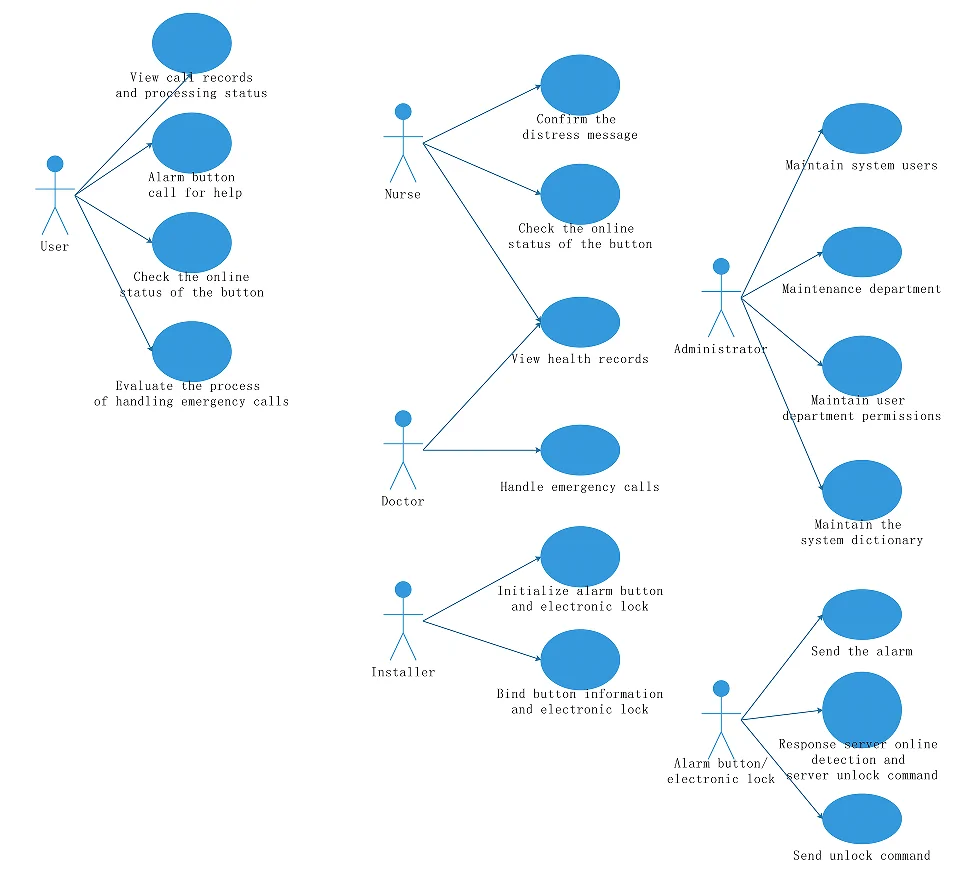
User use-case diagram of the emergency assistance system.

## Discussion

### Principal Findings

This implementation report describes the development and testing of a smart emergency assistance system designed to support rapid emergency response for older individuals living alone. The system successfully integrated IoT devices, cloud-based coordination, and hospital rescue workflows, enabling rapid detection, communication, and response under simulated real-world conditions. This study extends the concept of “emergency response” from alert generation to a full-process closed-loop workflow, proposing the “pre-entry access system” as a core functional module. Furthermore, it distinguishes between 2 progressive research phases—“simulation-based feasibility” and “real-world implementation”—emphasizing that the technology can serve as a feasible proof of concept for care practices for older adults in an aging society. From a practical standpoint, as described in the Introduction section, this study provides a prototype system that integrates alert triggering, multistakeholder response, remote physical access, and in-hospital data sharing, offering an operational solution to the physical access barrier that has been largely overlooked in previous research.

A principal finding is that the system achieved timely end-to-end emergency response, with distress alerts transmitted to the hospital rescue management platform within 5 seconds and confirmation feedback completed within 10 seconds. Beyond merely reporting these metrics, this finding implies that the MQTT-based communication framework and automated notification strategy are not only technically suitable for time-critical emergency scenarios but also operationally sufficient to support the additional coordination burden introduced by multistakeholder response and remote access activation—a requirement that simpler alert-only systems do not need to address.

Another key finding is the effectiveness of the pre-entry access control mechanism. By enabling remote unlocking of household entry points within 1 minute of rescue personnel arrival, the system addressed a commonly reported barrier in home-based emergency care–delayed access due to locked doors. Interpreted more broadly, this finding suggests that the next generation of emergency response products for older adults living alone must evolve from the “hardware+ call center” model toward an integrated framework of “hardware+ workflow integration+ physical access entry+ data coordination.” The pre-entry access mechanism demonstrated here is not merely a convenience feature but a critical determinant of whether golden window rescue efforts can proceed at all. The system also demonstrated stable operational performance, with reliable communication over distances of up to 30 kilometers and GPS-based positioning accuracy within 10 meters. These technical achievements, while important, are secondary to the core insight that a system designed for physical access integration can be embedded into existing hospital-centered emergency workflows without disrupting routine operations. Medical staff were able to process alerts, access patient health records, and coordinate rescue actions efficiently, suggesting that the additional complexity of managing remote entry does not impose an unacceptable operational burden.

Importantly, the simulated implementation showed that the system could be integrated into existing hospital-centered emergency response workflows without disrupting routine operations. Medical staff were able to process alerts, access patient health records, and coordinate rescue actions efficiently, highlighting the system’s compatibility with institutional practices.

Critically, unlike conventional personal emergency response systems (PERS) that primarily serve as alerting devices, the system described here functions as an end-to-end emergency treatment workflow engine, encompassing household entry, information acquisition, and multistakeholder coordination. This design represents a substantive shift from the “hardware+ call center” model toward an integrated framework of “hardware+ workflow+physical access+ data coordination.” In this implementation, the pre-entry access system was treated as an independent functional module, and multistakeholder synchronous response was embedded as a core design principle. The simulated validation approach proved to be an effective predeployment step, allowing for controlled testing of technical performance and process integrity prior to real-world piloting.

Collectively, these findings suggest that the system is technically feasible and operationally reliable in simulated emergency scenarios and that it has the potential to improve rescue efficiency and care coordination for older individuals living alone.

### Comparison With Prior Work

Previous studies and commercial solutions in the field of smart emergency response have primarily focused on wearable distress devices, fall detection systems, or emergency call services. While these approaches can facilitate emergency notification, they often do not adequately address downstream barriers, particularly the challenge of timely physical access to the patient’s residence.

The system described in this study extends prior work by explicitly incorporating an intelligent access control mechanism into the emergency response workflow. By linking emergency triggering with preauthorized, time-limited entry and hospital-side information integration, the proposed approach provides a more comprehensive implementation pathway. This design aligns with emerging perspectives in digital health implementation that emphasize end-to-end workflow integration rather than isolated technological components.

### Limitations and Lessons Learned

Several limitations should be acknowledged. First, direct system-level integration with the official emergency dispatch center has not yet been achieved due to the lack of an open data interface. As a result, emergency notifications currently rely on SMSs rather than fully automated dispatch integration. This limitation highlights the importance of institutional collaboration and policy-level support in the implementation of digital emergency systems. During the experiment, we also identified additional issues requiring further optimization, including that the hardware device casing and enclosure assembly have not yet been finalized, which may affect product maturity and reliability in practical use. Second, the current implementation was evaluated primarily through functional and simulated testing rather than large-scale real-world deployment. User acceptability, long-term system stability, and performance under high concurrency conditions require further validation. Third, regarding older adults’ digital literacy, the intelligent distress button has been designed with an antimisoperation mechanism—a single click is ineffective, and older users must press and hold the button for at least 3 seconds to trigger an alert. While this design reduces false alarms, it also assumes that users can understand and reliably perform this action during acute stress or cognitive decline, which may not hold true for all older adults, particularly those with severe cognitive impairment or motor dysfunction. Fourth, concerning user privacy and security, all system data are stored on servers on the hospital premises, and data transmission is encrypted through APIs. However, this architecture also implies that system functionality depends on continuous server availability and network connectivity within the hospital environment. Moreover, the current implementation has not undergone formal privacy impact assessments or security audits, which will be necessary prior to broader deployment.

From an implementation perspective, an important lesson learned is that technical feasibility alone is insufficient for successful deployment. System adoption depends not only on device performance but also on interoperability with existing health care infrastructure, clarity of workflows for emergency personnel, and trust from patients and their families.

### Implications for Practice and Future Research

The findings of this study have several implications for practice. For health care providers and emergency services, the proposed system demonstrates a feasible approach to reducing response delays in home-based emergencies through digital pre-entry authorization. For policymakers and system designers, the study highlights the need for standardized emergency data interfaces to support deeper integration between community-based technologies and official emergency dispatch systems.

Future research should focus on real-world pilot implementation and longitudinal evaluation to assess clinical outcomes, cost-effectiveness, and user acceptance. Additional work is also needed to strengthen data security, privacy protection, and system scalability, particularly in the context of large-scale deployment in aging communities.

### Conclusions

It should be explicitly acknowledged that the findings reported in this study were obtained from a simulated environment. In the fields of engineering and health services research, simulation-based validation represents a standard predeployment phase prior to real-world implementation. The value of this study lies precisely in providing controlled evidence that the system merits progression to real-world pilot testing. Deploying an unvalidated system directly into the home environments of real older users would entail high risks of failure and ethical concerns. Thus, the simulation-based approach was intentionally chosen as a rigorous and necessary preparatory step.

In conclusion, this study demonstrates the feasibility of an IoT-based intelligent emergency access control system designed to support rapid and coordinated emergency response for older individuals living alone. By addressing both communication and physical access barriers, the system provides a practical implementation pathway for improving home-based emergency care. With further optimization and institutional integration, the proposed approach holds promise for broader application in aging societies. Future research should focus on the following specific areas: (1) real-home pilot testing in actual households of older adults living alone; (2) assessment of acceptability among older adults, including those with cognitive impairment or technology-related fears; (3) safety validation through standardized testing protocols covering false alarms, missed alarms, device failures, and network interruptions; (4) exploration of multisensor fusion and machine learning algorithms to further reduce false alarm rates; (5) design of privacy-protective data collection and transmission protocols compliant with regulatory requirements; and (6) cost considerations, including low-cost alternatives and potential subsidy or insurance models to ensure affordability for the target population.

## Supplementary material

10.2196/91542 Multimedia Appendix 1 Overall architecture of the smart emergency assistance system.

10.2196/91542 Multimedia Appendix 2 Example of an emergency call button used in the proposed system.

10.2196/91542 Multimedia Appendix 3 Example of a system-generated emergency alert message.

10.2196/91542 Multimedia Appendix 4 Emergency medical services (EMS) confirmation of an emergency alert.

10.2196/91542 Multimedia Appendix 5 Interface for accessing patient health records in the rescue management platform.

10.2196/91542 Multimedia Appendix 6 The preparation steps for clinical application include the following key steps.

## Abbreviations

EMS: emergency medical services
IoT: Internet of Things
LED: light-emitting diode
MQTT: Message Queuing Telemetry Transport
PERS: personal emergency response systems
QoS: Quality of Service
TLS: Transport Layer Security

## References

[1] (2024). Statistical communiqué of the People’s Republic of China on the 2024 national economic and social development. National Bureau of Statistics of China.

[2] Li ZH, The Drafting Group of General Report (2015). The general research report of Chinese strategic for dealing with population aging [article in Chinese]. Sci Res Aging Mar.

[3] Niu D (2024). A study on health care for elderly people living alone: from the perspective of collaborative governance [Article in Chinese]. Int Public Relat Oct.

[4] Ma J, Li L, Geng HB (2019). Construction of a three-level emergency response information collaboration system for smart home-based elderly care services [Article in Chinese]. Libr Inf Serv.

[5] Dong JN (2024). Development of a high-precision wearable health monitor for infants and young children [master’s thesis].

[6] Silva J de C, Rodrigues J, Al-Muhtadi J, Rabêlo RAL, Furtado V (2019). Management platforms and protocols for internet of things: a survey. Sensors (Basel).

[7] Wu HF, Li SD, Zhu GY (2025). Control system design for ankle rehabilitation robot based on ESP32 [Article in Chinese]. Techn Autom Appl.

[8] Sun Z, An T, Zhang DD (2024). Design of remote detection system based on ESP8266 microcontroller [Article in Chinese]. SmartTech Innov.

[9] Quan LL (2024). Research on product design of hospital aging-adapted self-service terminal. [master’s thesis].

[10] Yuzhu F, Jiannan W, Yuxin M, Xia L (2023). IoT-based wearable devices and vital signs monitoring system. Ind Technol Innov Oct.

[11] Zhang L, Jiang YB, Zou LH (2021). Arduino-based smart home medicine cabinet. Sci Technol Innov.

[12] Hu ZZ, Zhang ZK (2024). Application of UML model in information system requirements analysis. Mod Comput Jun.

