# Supplementary material for: Smart Emergency Assistance System With Pre-Entry Access for Older Adults Living Alone: Simulation-Based Implementation Report
Source: JMIR Form Res. 2026 Oct 23;10:e91542. doi: 10.2196/91542 (PMC13717330; doi:10.2196/91542)
Supplement: Supplementary file 4 [file formative-v10-e91542-s006.docx]

**The preparation steps for clinical application include the following key steps**

①Equipment installation and initialization: Install a smart key box at the user's doorstep and lock the entry key inside. At the same time, install and initialize the smart distress button and smart key box at home to ensure that the two devices are connected to the network and complete the information binding within the system.

②Emergency triggering: In the event of an unexpected injury emergency, the caller only needs to press and hold the call button for 3 seconds or more to trigger the alarm.

③Alarm information transmission: The built-in communication system of the distress button will immediately send the distress message to the hospital rescue management platform and provide feedback to the caller through the illuminated light, confirming that the alarm has been successfully sent.

④Platform response and confirmation: After receiving the distress message, the hospital rescue management platform automatically notifies medical staff to confirm. The system immediately notifies 120 to go for rescue and notifies the family members of the caller based on the reserved contact number. Medical staff view patients' historical diagnosis and treatment information based on their hospital health records.

⑤Quick entry and on-site signal: If a password key box is installed, the platform automatically sends the reserved 6-digit key box password to the rescue personnel's mobile device. If an electromagnetic key box is installed, the platform actively remotely controls the key box to automatically unlock, allowing rescue personnel to quickly enter the scene. At the same time, the key box emits an SOS Morse code flashing on-site distress signal, making it easier for neighbors and other personnel to assist in rescue efforts.

⑥Emergency preparedness of the hospital: The hospital synchronously carries out emergency preparedness for patients upon admission, and prepares corresponding rescue equipment and drugs based on the patient's condition and historical medical information in the "health file" query.

⑦Real time monitoring and feedback: During the entire rescue process, emergency contacts can view the progress of medical rescue in real time through the mobile web platform, ensuring that family members can timely understand the patient's rescue situation.
