# Supplementary material for: Smart Emergency Assistance System With Pre-Entry Access for Older Adults Living Alone: Simulation-Based Implementation Report
Source: JMIR Form Res. 2026 Oct 23;10:e91542. doi: 10.2196/91542 (PMC13717330; doi:10.2196/91542)
Supplement: Supplementary file 8 [file formative-v10-e91542-s001.doc]

**Overall architecture of the smart emergency assistance system.**
